# Influence of ADAPTIL^®^ during the Weaning Period: A Double-Blinded Randomised Clinical Trial

**DOI:** 10.3390/ani10122295

**Published:** 2020-12-04

**Authors:** Natalia R. Santos, Alexandra Beck, Cindy Maenhoudt, Alain Fontbonne

**Affiliations:** 1 Unité de Médecine de l’Elevage et du Sport (UMES), Ecole Nationale Vétérinaire d’Alfort, 7 Avenue du Général de Gaulle, 94700 Maisons-Alfort, France; cindy.maenhoudt@vet-alfort.fr (C.M.); alain.fontbonne@vet-alfort.fr (A.F.); 2 Ceva Santé Animale, 33500 Libourne, France; alexandra.beck@ceva.com

**Keywords:** behaviour, dogs, weaning, maternal, pheromone, introduction

## Abstract

**Simple Summary:**

The process of weaning can potentially affect the development of dogs due to frustration of both the dam and the puppies and, consequently, affect their interactions. Comprehending the dam and puppies’ interactions during the weaning process could provide information to help overcome the challenges of this period. In addition, the use of ADAPTIL^®^, a dog-appeasing pheromone, could potentially reduce the stress and decrease the frustration associated with weaning. To better understand the dam and puppies’ interactions around the weaning time and the effect of ADAPTIL^®^, 25 bitch/litter dyads were evaluated under the influence of ADAPTIL^®^ (*n* = 14) or a placebo (*n* = 11). Video recording allowed the evaluation of the dam and puppies’ behaviours at weeks three/four, weeks five/six and weeks seven/eight). Contact of the dam and the puppies were inversely affected when compared to puppy-to-puppy interactions. Over time, the dam spent less time with the puppies, and the puppies played more often with their littermates. Under ADAPTIL^®^, the puppies seemed to cope better with frustration, and the bitches were more relaxed in the presence of the puppies. The perception of the breeders measured by visual analogue scales indicated a beneficial outcome of the use of the pheromone in the dam/puppies relationship during the weaning period.

**Abstract:**

ADAPTIL^®^, a dog-appeasing pheromone, was shown to modify the dam–puppies’ interactions during the neonatal period but could also influence the weaning period. Fourteen bitch/litter dyads continuously exposed to ADAPTIL^®^ from the third/fourth weeks until the seventh/eighth weeks postpartum were compared to 11 dyads exposed to a placebo. Maternal and puppy behaviours were video-recorded, and at three time points (weeks three/four, weeks five/six and weeks seven/eight) after parturition. The well-being of the puppies and the overall relationship with the bitch were assessed using visual analogue scales (VAS) completed by the caregivers. All mothering behaviours, such as time of contact, licking and the amount of time dedicated to nursing puppies, decreased gradually from weeks three/four to weeks seven/eight. A switch in nursing position was observed over time: the use of the standing position increased compared to the lying position. The treatment had an effect on the nursing position: bitches in the ADAPTIL^®^ group nursed more often in a lying (*p* = 0.007) or sitting position (*p* = 0.037), whereas for the placebo group, they favoured the standing position (*p* = 0.011). Once the puppies became more demanding for suckling, the bitches started showing rejection signs or aggressive growling, with a peak at weeks seven/eight. The pheromone seemed to reduce the intensity of avoidance in bitches exposed to ADAPTIL^®^ at all time points. The score of all events combined as a sign of frustration showed a difference over the full period (*p* = 0.003), with the placebo group having a significantly higher score. From the caregiver perspective (through the VAS), under ADAPTIL^®^, the bitches were calmer when puppies tried to suckle (*p* = 0.001), more tolerant towards pups (*p* = 0.025), showed a greater motherly attitude (*p* = 0.016), the puppies cried less when left alone (*p* < 0.001) and interactions amongst pups were more harmonious (*p* = 0.055). Under ADAPTIL^®^, the bitches were less annoyed by the puppies, who seemed to cope better with frustration. The breeders perceived a benefit of the pheromone during the weaning period.

## 1. Introduction

The maternal care in dogs has been proven to have a direct impact on puppy development. Recent studies have pointed out how critical and important it is to comprehend the effect of maternal behaviours [1,2,3]. The interval between birth and weaning in dogs is brief but is also an intensive period of changes, development and adjustments to prepare the puppies for greater independence in the postweaning period. In general, during this window, puppies will go through two important periods [4,5]. The “neonatal” period begins around the third day of life and ends on about day 16, followed by the “socialisation” that goes from the third week until the puppy reaches 12–14 weeks of age [5]. Both of these phases potentially represent a high-risk zone for abnormal development that could have a long-lasting influence on the puppy’s behaviour. However, although the neurological development of puppies has been well-described during these periods, studies focusing on the behaviour from 21 days postpartum to weaning are scarce [6].

The development of puppies during these first weeks is rapid and intense. Changes in the way of moving, improvement of their coordination, ability to maintain their own body temperature and the opening of the ear channels and eyes followed by self-control of urination and defecation lead to more independence from the dam [7,8]. From weeks three to 12, the pups start to display several adult behavioural patterns [9]. During this time, puppies begin to recognise their conspecifics and other social networks, which would include humans and other dogs. In relation to food consumption, by the third week, the puppies should be able to start the transition to voluntary consumption of moistened solid food, which is one of the first steps of the weaning process. This transition time seems to bear an effect on the puppy’s development. Studies have demonstrated that early weaned puppies—at four–six weeks—are more prone to behavioural problems when compared to puppies with a normal weaning time [10,11,12]. The negative effects on the behaviours of adult dogs due to a short weaning time [12] give some insight of possible areas of early intervention to improve puppy development.

A better understanding of the dam and puppies’ interactions during the weaning process could facilitate overcoming the challenges of this period. Weaning starts with the introduction of another source of food besides milk from the dam and ends with a complete arrest of nursing. The combination of the reduction of milk production normally ending between seven to 10 weeks after giving birth [4] and the increase in solid food intake of puppies occurs at the same time as the decrease in nursing activity and, eventually, weaning. Besides the nutritional aspect, it is important to consider that suckling also seems to be a source of psychological satisfaction in social animals like the dog [13]. When another source of nutrition is introduced to the puppies, the dam makes herself less available for the puppies to nurse. Amongst different strategies, the dam adopts a body position that renders access to the teats difficult [14] or keeps a distance from the puppies [15,16]. Dams also start being aggressive towards the puppies, most likely as a reaction to the pain felt when they try to suckle with their small, sharp teeth. This natural and gradual process is the first step of discontinuing a puppy’s maternal bond to encourage greater independence and exploratory behaviours. Concurrently, the weaning period can create frustration that all need to overcome. As defined by McPeake et al. [17], frustration is a negative emotional reaction experienced after a given expectation is violated, which happens for both the dam and the puppies at weaning time.

Our aim in the current study was to categorise the variations of maternal and puppy behaviours from three weeks after birth until the weaning time and the effect of a dog-appeasing pheromone product (ADAPTIL^®^ Ceva Santé Animale, Libourne, France) during this period. We observed whether the use of ADAPTIL^®^ had an effect on interactions between the dam and her puppies and among the puppies to help them adjust during this critical period. ADAPTIL^®^ is intended to reproduce the natural dog-appeasing pheromone released by the bitch from the mammary zone after whelping [18]. ADAPTIL^®^ is clinically proven to help dogs feel calm and relaxed in stressful situations [19,20,21,22,23,24,25,26,27]. It provides a strong signal of comfort and security to the puppies but, also, has the same effect on dogs of all ages. In a previous study [28], we assessed the benefits of ADAPTIL^®^ on the maternal behaviour of the bitch and the interactions with the puppies in the peripartum period. In this study, we hypothesised that ADAPTIL^®^ could lead to further behavioural differences around weaning. Signs of bitches being comfortable and relaxed while the puppies cope well with the frustration possibly associated with suckling cessation and their mother being less available could reflect these differences, helping both bitches and puppies to better adjust to the weaning process.

## 2. Material and Methods

### 2.1. Subjects

This multicentric field trial aimed at recruiting at least 24 bitches and their respective puppies. The target was to enrol a minimum of 12 bitches and their litters in each group. They were recruited from professional breeders in France through the client database of the Centre d’Etudes en Reproduction des Carnivores (CERCA), 94700 Maisons-Alfort, France and the list of breeders registered with the French Kennel Club. The conditions of weaning were specific to each kennel, respecting the following inclusion criteria. In relation to the breeding kennels, breeders were required to produce at least 8 litters per year and begin to introduce solid food around the third week of age. The mortality rate between birth and weaning had to be lower than 10% (based on the breeders’ records). Based on the number of breeding bitches, the selected kennels were characterised as small (less than 10 bitches), medium (10 to 20 bitches) or large (more than 20). The housing system required each litter to be separately housed in a well-defined weaning area, where puppies lived together from the beginning until the end of the trial (7/8 weeks postpartum). This was the same housing where bitches whelped and were continuously living with their litters for the three weeks preceding the start of the current study (maternity area). Two housing systems were represented in the trial. A single maternity was defined when the bitch and the litter (dyad) were housed in an isolated room. In the collective maternity, the bitch and the litter were housed in individualised areas separated by walls in a common room. In both systems, at weeks (W)3/4, the puppies had a delimited area defined by the whelping box or a small, movable wall allowing the dam to be physically separated from the puppies, although the bitch was inside the maternity area. At W5/6, the puppies could move more and had access to the whole area. During the weaning process, the dam was allowed to be outside the maternity area during the day. This common practice by the breeders facilitates weaning but, also, most likely causes frustration to the puppies, due to separation from the dam. The time of separation in total was no longer than 8 h/day to homogenise the conditions between litters. The selection of the bitches was independent of parity, but only mesocephalic or dolichocephalic breeds (based on the calculated cephalic index [29]) were selected. The groups of bitches were classified as small (<15 Kg), medium (15–25 Kg) and large (>25 Kg) [30]. In addition, each selected bitch was required to have a litter of at least three healthy puppies at 21 days postpartum to allow a minimum level of interactions between puppies. The bitches were not allowed to have been previously exposed to a dog-appeasing pheromone product (ADAPTIL^®^) during the current pregnancy. In most of the cases, the bitches and their litters remained in the maternity/weaning area from 21 days postpartum until the end of the study. For those transferred to another area at 5 to 6 weeks postpartum to allow more space for the puppies, the area was pre-exposed to the treatment one day before the transfer. Enrolled bitches were not exposed to any product likely to modify behaviour other than the one tested. Reproductive history and parturition data were collected when the bitch entered the trial. During the trial, each dam was kept with her own puppies, and no interactions amongst different litters were allowed. The impact of the proximity of another bitch (single or multiple housing) on the behaviour of a bitch and her litter were not taken into account during the data analysis. The study was based mainly on the observation of video footage of the puppies and the dams; no direct contact between the observer and the animals was necessary, and the routine of each kennel was not modified. Due to the nature of the study, no ethical approval was requested.

### 2.2. Treatment

Treatment was provided via a plug-in diffuser device releasing a dog-appeasing pheromone product or a placebo contained in completely identical refill vial. Only the ID number differed between treatments. The diffusers were placed at the first observation time (3/4 weeks postpartum) and spread constantly in the environment until the end of the trial. Allocation of treatment was randomised and double-blinded. All personnel involved in the study were blinded to the treatment conditions (the study investigator, the breeders and their staff and the statistician). The only exception was the sponsor representative, who allocated treatments according to a randomisation list while ensuring there was no concurrent use of both treatments (ADAPTIL^®^ and placebo) in the same kennel.

A single diffuser was used in individual areas or in the maternity area if it was smaller than 70 m^2^, according to the product’s manufacturer’s instructions. Based on the information provided by the sponsor representative, each diffuser, identified with a single numeric label, was plugged in at a height of around 1.5 m above the floor to optimise the diffusion. Two refill vials (A and B) of the same ID number were provided to cover the full study period. The first refill was plugged in at the first day of enrolment (at 3 to 4 weeks postpartum) by the study investigator and replaced by the second refill at 5 weeks after parturition. All diffusers were removed at the end of the study at 7/8 weeks postpartum.

### 2.3. Data Collection

Primary outcome measures consisted of behaviour assessments of both the dam and the puppies, separately. Secondary outcome measures consisted of activity levels.

#### 2.3.1. Maternal and Puppy Behaviours

Two methods were used to assess maternal care and puppy behaviours. Three time points were chosen to observe the behavioural evolution of the dam and the puppies during the postpartum period until the completion of the weaning process. The first visit occurred at three or four weeks (W3/4), the second one at five or six weeks (W5/6) and then the last one at seven or eight weeks (W7/8), respectively.

The first method was a video recording of maternal and puppy behaviours. During each 2-week period, a single video recording was collected, with a target duration of at least two hours.

Recordings were performed as much as possible during a quiet time in the kennel (either early in the morning or at lunchtime) and when human presence was limited to reduce any interference in relation to the behaviour of the dam and puppies. During part of the recording or the total duration of the video, dams were present, with the exception of a German shepherd bitch who was removed from the puppies (for travel reasons) before the last evaluation at W7/8. The cameras were attached to the wall in close proximity to the animals to guarantee a good visualisation. Due to the complexity and variety of the behaviours observed, the videos were analysed using a logging software for video/audio coding called Behavioural Observation Research Interactive Software (BORIS) [31]. BORIS integrated the possibility of playback with the time offsets of media files, allowing to transcribe the ethogram previously defined in an easy way. The experimental unit was the mother and her puppies. In the current study, two types of events were evaluated: the state events where behaviours were measured in duration (minutes) and the point events where the behaviours were measured as a frequency (Table 1). For maternal behaviours, the focus was the dam and the interactions with her puppies. The variables measured were based on the list of maternal behaviours described in the literature [1,2,14,32,33]. Some are positive (expected maternal behaviours) such as body contact or nursing. Others may reflect a more negative attitude, such as avoiding puppies (through rejection signs and/or aggressive growling). For puppy behaviours, the focus was the behaviour itself and all dyads (to the dam or other littermates). The point events defined for the dam and for the puppies attempted to reflect different possible reactions to the potentially distressing weaning period.

If the bitch was introduced or entered the maternity area when the video was being recorded, the time until puppies approached the mother was measured as the latency time. Similarly, if the bitch left the area during the video recording, the time puppies stayed at the door, looking for her return was also recorded.

The method of continuous sampling was used. Since the experimental unit was the mother and her litter, puppies were not individually identified but assessed all together, with all behaviours of each puppy considered at each time point. To take into account the different lengths of all individual recordings, all behaviours were expressed as a percentage of the recorded time per session. To reduce the chances of errors, each video was analysed separately to evaluate maternal and then puppy behaviours.

The second assessment method was through visual analogue scales (VAS), a commonly used tool to measure subjective outcomes in humans [34] and veterinary medicine [35], such as in pain management or dermatological signs assessment, and previously described by Santos et al. [28]. Each VAS (seven in total) consisted of a question answered through a small mark placed on a 10-cm line whose ends corresponded to extreme attributes (negative and positive outcomes). The same person, a caregiver in daily contact with the litter, completed the scales on the same day as the video recording. Four questions were associated with the puppies in relation to food (if the weaning food was eaten well), signs of diarrhoea, puppy behaviour in the absence of the dam and interactions between puppies (signs of competition for access to the bitch or to the bowl of food by shoving and growling). The three other questions focused on the dam, evaluating if the bitch allowed the puppies to nurse without resistance, if any signs of aggression towards the puppies were observed and the overall relationship between the bitch and her litter (spending time in interacting with them). The numerical score for each question was calculated based on the length (in mm) from the left end to the mark (from 0 to 10), a zero reflecting the most “negative” answer for each parameter and a 10 score the most “positive” situation.

#### 2.3.2. Activity Tracker Data Evaluation

To record the bitch’s activity profile, an electronic activity tracker device (Fitbark^®^, Kansas City, MO, USA) was attached to the collar at enrolment until the end of the trial. Individual data was uploaded and synchronised to a smartphone through the Fitbark application. The daily information retrieved was divided into three main categories as “play”, “active” and “rest”, corresponding to high activity, medium activity and no movement (standing still or sleeping), respectively. “Play”, “activity” and “rest” were analysed for each individual animal. Then, “active” and playing” times were combined into a single “active play” parameter, as opposed to “rest”, since it was more meaningful to group those parameters to better reflect the bitches’ overall activity. In the Results section, “activity” will refer to “active play” time.

#### 2.3.3. Statistical Analysis

Variables were described on the total population and by treatment group, by time point and on the full study period (all times combined). Quantitative variables were described, and boxplots were plotted to notice potential outliers (none were detected). The normality of data was checked for each group by a Shapiro-Wilk test (*n* < 50) for an alpha level equal to 1%. If the assumption of normality was met in both groups, a Student’s *t*-test (parametric) was performed by considering equal or unequal variances. If normality was not observed for at least one group, a Wilcoxon test (nonparametric) was then performed. For qualitative variables, a chi-square test or an Fisher’s exact test was done at the 5% level, depending on the number of observations in each category.

Models were then performed as follows: for video criteria and VAS related to bitches, the response was considered to be the result of the treatment group, the time point, the group*time point interaction, the parity (primiparous versus multiparous) and the number of puppies in the litter. For video criteria related to puppies, in addition to the above criteria for bitches, the proportion of time the bitches spent inside the maternity area with their puppies (as opposed to the time they were out) was also taken into consideration in the models. Indeed, bitches could exit the weaning area (where camera was recording) for daily walks or physiological needs, and puppies could be alone in the indoor area for some minutes. Global effects for each parameter and comparisons between each group at each time point were calculated. Models were performed either on raw data or on transformed data (ranks) according to the residual study results (error model term). If the normality was met and residuals were homogeneous, then results were presented on the raw data. In all other cases, analyses were made on ranks and comparisons with the raw data results. If both results were consistent, then the raw data were presented; otherwise, the rank data were kept. Significant threshold was fixed at 5%.

## 3. Results

Twenty-seven bitches and their respective puppies were recruited from eight professional breeders and completed the study. Selection and follow-up extended from February to June 2019. Inclusions were stopped when the target sample size was reached (three additional bitches were recruited, to anticipate possible postinclusion exclusions). Two bitches from the same kennel were excluded a posteriori due to missing data (only data from two time points were recorded). Of the twenty-five bitches that were analysed (Table 1), 14 were exposed to ADAPTIL^®^ (for a total of 76 living puppies at day 21) and 11 to the placebo (for a total of 88 puppies). All eight facilities were allocated to one single treatment, with all their selected bitches exposed to either ADAPTIL^®^ or placebo (Table 2). Exposure to the diffuser in the weaning area started from at least the third week postpartum (in some cases, it started at the end of the second week), and bitches and their litters were continuously exposed to the product until the end of the trial. The different breeds were randomly allocated to treatment groups. Only one breed (Golden retriever) was present in both groups but accounting for a small number of bitches (one and two, respectively). Overall, in relation to breed size, only large-size breeds were represented in the placebo group, while the treatment group had 21.4% of small (3/14), 35.7% of medium (5/14) and 42.9% of large (6/14) breeds. In terms of weaning practices, all selected facilities introduced moistened solid foods from the third week, while ensuring that bitches were out of the area to encourage the puppies to eat and avoid competition between the mother and her puppies. Bitches were still with the puppies until at least seven weeks of age—thus, for all assessment times—with the exception of a German shepherd bitch that was removed from the puppies (for travel reason) two days prior to the puppies reaching seven weeks of age.

Both groups were well-balanced regarding the age of the selected bitches (mean age = 3.8 ± 1.8 in placebo and 3.1 ± 1.3 in ADAPTIL^®^) and parity (four and four primiparous and seven and 10 multiparous, respectively; *p* = 1). Overall, more multiparous bitches (*n* = 17, 68.0%) than primiparous (*n* = 8, 32.0%) were enrolled in the study (Table 2). Multiparous bitches had produced one to six previous litters prior to this study and no previous abnormal maternal behaviours reported by their breeders. No difference was found between groups (*p* = 1) regarding the general temperament of the bitches, as qualified by their breeders. In total, sixteen bitches (69.57%) were considered as rather calm versus seven perceived as rather nervous (30.43%).

For the placebo group, three kennels were defined as medium sizes (75%) and one small (25%), while for the ADAPTIL^®^ group, two were medium-sized structures (50%), one large (25%) and one small (25%). Bitches in individual maternity areas was the most common housing for the ADAPTIL^®^ group (Table 2). Collective maternity units were more frequently used in the placebo group, although 62.5% of placebo bitches (five/eight) housed collectively were transferred to single individualised boxes at weeks five/six to allow more space for the puppies. On average, bitches were separated from their puppies two to three times a day, accounting for a mean total daily separation time of 62.8 min at week three (i.e., about one hour), 184.2 min (3 h) at weeks five/six and 302.5 min (5 h) at weeks seven/eight.

### 3.1. Video Observation

A total of 196 h, 32 min and 45 s of footage for the mother–puppy dyad were viewed multiple times and analysed by one single observer. The overall length of collected video recordings for each bitch and her litter were between 00:46:00 (one single video due to unintentional switch-off of the camera) and 05:00:18, with an average length of 03:04:20 at week three to week four, 02:36:52 at week five to week six and 02:23:39 at week seven to week eight. To take into account those differences, data was measured either as a duration or as a frequency, and the analysis was done in relation to the percentage of the total video-recorded time. Bitches (both groups combined) spent, respectively, 90.5% of the time inside the weaning (maternity) area with their pups at week three to week four, 93.1% at week five to week six and 70.9% at week seven to week eight. To take into account possible differences in the proportion of time bitches were out of the weaning area during the recordings (for those having free access outside), the analysis on the video parameters focusing on the puppies considered this parameter.

- Maternal Care

The amount of time dedicated to nursing puppies decreased from weeks three/four to weeks seven/eight, which is consistent with the introduction of solid food. Differences were observed between each time point, with bitches nursing longer at weeks three/four and then decreasing continuously (Table 3). There was a tendency for bitches in the placebo group to spend more time nursing (*p* = 0.12, all periods mixed). Over the whole period, when one puppy started to suckle, the others quickly joined in. Most of the time, the dam nursed the whole litter at once.

An evolution in terms of the nursing position was observed (Table 4), with bitches nursing mainly in a lying position when puppies were younger (79% of the time at weeks three/four) and switching to a standing position as the puppies grew (70.1% of the time at week seven to week eight). Lying down and standing positions evolved in opposite and complementary ways, while sitting was negligible and consistent over time (11–13%).

Over the full weaning period, a significant treatment effect was found (Table 5). Bitches exposed to ADAPTIL^®^ nursed puppies in a lying (*p* = 0.007) or sitting position (*p* = 0.037) more often, while placebo bitches nursed significantly more in a standing position (*p* = 0.011). Moreover, bitches in the ADAPTIL^®^ group preferred the lying posture to the standing one, despite the reversal of posture (lying versus standing) observed over time, whatever the treatment group.

The behaviour of licking the puppies decreased with time regardless of treatment, bitches licking their pups on average 4.7 times per hour at weeks three/four, 2.8 times per hour at weeks five/six and 2.6 times per hour at weeks seven/eight. Based on the comparison estimates between groups, bitches tended to lick their puppies more frequently in the ADAPTIL^®^ group than the placebo group at each time, regardless of the litter size (number of puppies effect: *p* = 0.87).

- Puppies’ activities

Sleeping was the main activity of puppies, representing 89.7% of their time at weeks three/four, 82.8% at weeks five/six and still 71.8% at weeks seven/eight. No differences between groups were observed, as expected, on this physiological parameter. The proportion of time interactions between mothers and puppies (playing, providing care, walking around, etc.) also decreased over time, from 38.3% at weeks three/four to 21.4% at weeks seven/eight as the puppies developed and became more independent. In parallel, interactions between littermates increased, from 9.3% of the time of video recordings at weeks three/four to 16.6% at weeks five/six and 17.1% at weeks seven/eight (Table 6). No case of puppy isolation was observed during the trial.

- Reaction to separation

Beyond the care provided to pups, the main goal of this study was to assess the behavioural aspects of the weaning transition, both in the puppies and the bitches, and, in particular, how puppies adjusted to the separation from their mother and her attempts to make them more independent. The length of separation between mothers and litters gradually increased throughout this period, as previously mentioned. Puppies’ reactions when the bitch left the room, measured by the time they were looking for her and staying close to the door waiting for her return, gradually decreased over the study period (Table 6). Conversely, when the dam returned, puppies from both groups tended to promptly search for the bitch but with a decrease again between weeks three/four and weeks seven/eight, as they became more mature. The latency time, as defined by the time for puppies to approach the mother when the dam returned to the maternity area, was a little longer in the ADAPTIL^®^ group than in the placebo group at weeks three/four and weeks five/six (respectively, 5 and 4 s more). Although not significant (respectively, p = 0.28 at weeks three/four and p = 0.26 at weeks five/six) this difference suggests that puppies might be slightly less distressed by the absence of the bitch, but this would need to be confirmed on a large sample.

As puppies grow up, bitches tend to become less available for them or even reject them, creating potential frustration and anxiety. Several behaviours were assessed to estimate if and how these emotions were being experienced. Amongst them, rejection signs from bitches towards their pups were observed to increase from the beginning (0.3 times per hour in the weeks three/four) to the end of the weaning period (up to 2.9 times per hour on average at weeks seven/eight). Only three bitches (from the placebo group) never displayed signs of rejection towards the puppies during the trial. Rejection signs were observed only in 16% (4/25) of the bitches at weeks three/four (all from the control group) and then in 56% (14/25) of all the dams at weeks five/six, which increased to 76% (19/25) at the last observation. Similarly, aggressive growling from bitches toward their pups increased from 0.2 times per hour in weeks three/four to up to seven times per hour on average at weeks seven/eight. In order to suppress suckling from their puppies, bitches displayed aggressive behaviours. “Punishment behaviours” (aggressive growling and rejection signs) were variable amongst the bitches and appeared for the first time at weeks three/four in 16% of bitches (4/25).

The frequency of aggressive growling also varied, increasing to 64% at weeks five/six and then to 68% at weeks seven/eight. Two bitches in each group never showed signs of aggression towards the puppies (a Labrador and a Golden Retriever in the placebo group and two German Shepherds in the ADAPTIL^®^ group). Due to a great variability in relation to the frequency of each isolate behaviour, a composite criterion was designed combining the rejection signs, lip-licking and aggressive growling towards pups to better assess the bitch’s “irritability” level. This criterion was found to drastically increase from 3.5 times per hour at weeks three/four to up to 16.5 times at weeks seven/eight, reflecting bitches’ intolerance when solicited too frequently by their pups as they grow. Although not significantly different (*p* = 0.8 during the full study period), bitches exposed to the placebo displayed slightly more irritability (as assessed through this score) than bitches exposed to the pheromone treatment, whatever the time point.

Similarly, a composite criterion was defined to assess the frustration reactions by the puppies when separated or even rejected by their mother, since no differences were observed in the analyses of single behaviours. This global score combined yawning, lip-licking, scratching (displacement behaviours known to potentially reflect discomfort or even anxiety [27]), barking, growling and searching-mother signs. This criterion showed no significant differences between groups at each time point, but the differences were significant during the full study period (*p* = 0.003), with more frustration reactions and anxiety signs overall displayed by the puppies exposed to the placebo as compared to the ADAPTIL^®^ group. Moreover, based on the comparison estimates between groups, these differences were confirmed regardless of the litter size discrepancy between groups (no impact of the number of puppies effect: *p* = 0.37).

### 3.2. Breeders’ Perception of Maternal Care

The visual analogue scales (VAS) highlighted interesting evolutions over time (from weeks three/four to weeks seven/eight) on the full study population, regardless of treatment (Table 7). While motherly attitudes stayed globally equal, according to the breeders, the mothers’ tolerance to pups decreased over time, especially when solicited for nursing as puppies grew. The maternal interactions were more peaceful at weeks three/four when the puppies were less mobile, then more conflictual around weeks five/six, most likely due to the puppies searching to suckle, and then slightly improved at weeks seven/eight as the puppies learned the limits imposed by the dams.

No effect of the treatment was observed, time-by-time, on the maternal behaviours of bitches and the puppies’ behaviours, as assessed by their breeders. However, on the global weaning period (all three times combined), all VAS criteria showed significant differences favouring the bitches and litters exposed to ADAPTIL^®^ over the placebo (Table 8). The analysis of the full study period, including weeks three/four, since the diffusers were placed before this time, allowed conclusions on the global benefits of the weaning time. The number of puppies did not influence the VAS results for maternal or puppies’ parameters. The influence of number of puppies when a litter size was a covariate was not significant for the mother (*p* = 0.96, 0.09 and 0.56) nor for the puppies’ parameters (*p* = 0.96, 0.77 and 0.44). The parity of the bitches only had an impact on “bitch global relationship to puppies”, with higher mean scores in primiparous bitches (both groups combined, mean = 9.3 ± 1.1) than multiparous (mean = 8.0 ± 1.9) (*p* = 0.007).

### 3.3. Activity Tracker

The Fitbark device was well-tolerated by all animals, with no complications or issues being reported. The recording of the data was less reliable than formerly published [28]. The system failed to synchronise, with data missing from six bitches (24.0%) and a partial loss from six other bitches (considered as missing data). There were no significant differences observed between the groups in terms of daily activity level. Bitches in the ADAPTIL^®^ group tended to rest a little more during the weaning period, on day (D)21–D49 (mean “resting time” = 12.2 (±0.9) hours per day), than those exposed to the placebo (mean = 11.7 ± 1.2 h).

## 4. Discussion

This study assessed a dog-appeasing pheromone treatment, compared to a placebo, in bitches and their puppies during the weaning period (from three weeks until seven/eight weeks postpartum). We observed that the use of ADAPTIL^®^ seemed to have a positive impact on both the bitches and the litters to help them adjust during this critical period.

Although the study investigator, breeders and their staff and the statisticians were blinded towards the treatment group, the treatment allocation was partially controlled by the study representative in order to prevent the test product and placebo being concurrently released in a same facility at the same period. Including bitches from the same environment, most likely the same breed, raised and assessed by the same person would have provided a nice opportunity to control for those variables and allow comparisons between groups within a same kennel. However, this would have required a minimum three-week washout period before enrolling new bitches allocated to the other treatment to ensure sufficient time for the cleaning of the surfaces and complete dissipation of pheromone traces. The length of follow-up in the current study (six weeks) and the available time for the clinical project did not allow this option to be considered.

Since no similar data was available and this study was exploratory, no sample size calculation was made by the statisticians. The empirical target was to recruit at least 12 bitches and their litters in each group, which was reached, with 27 bitches recruited. Two had to be removed from the analysis, leading to a sample of 25 bitches and their corresponding litters. The data were collected in two-week periods (week three to week four, week five to week six and week seven to week eight) for convenience in the schedule of the breeder’s visits from the study investigator. This implies that, within each treatment group, there were puppies of different ages, with some puppies one week older than the others. However, this was the case for all puppies from both groups and whatever the study visit. On average, due to random allocation, these differences were supposed to be balanced between both groups.

The possible impact of the proximity of another bitch on the behaviour of a bitch and her litter were not taken into account during the data analysis due to the small sample size of those subgroups in each treatment group. However, the distribution of housing conditions (single or multiple) was balanced most of the time. The only exception was at weeks three/four (when bitches spent most of their time nursing their puppies, and puppy behaviours were mostly dedicated to eating and sleeping), when more single housing was observed in the ADAPTIL^®^ group (78.6%) versus more collective housing in the placebo group (72.7%). From weeks five/six until the end of the study, a transfer occurred, and groups were perfectly balanced (78.6% and 72.7% of the bitches, respectively, in both groups being housed individually), allowing more space for bitches and puppies to develop and display their full behaviour potentials. Due to the configurations of the rooms (separated), no impact of the neighbour animals was expected.

For practical reasons (the study investigator having to visit many different breeding facilities located far away from each other in France), and to try to minimally disrupt the breeders’ schedules and habits, it was not possible to collect baseline data before plugging the diffusers and then recollecting the first dataset in the same facility. Different breeds were selected in each group, with larger-size breeds represented in the placebo group. As previously described [36], this difference might have affected the litter size discrepancy between groups, which was significantly greater (*p* = 0.004) in the placebo than the ADAPTIL^®^ group. To increase the accuracy of the results, the effect of litter size was integrated as a covariate in the statistical models, since it was an unwanted difference between the groups.

A minimum of at least two hours of continuous recording was collected for each bitch–litter dyad at each time point. This duration, despite providing a heavy amount of data to analyse, might not be representative of the full behaviour of the animals. The decision to set the video observation to at least two hours for three specific times during the weaning period was guided by the objective to compare two treatments and not to fully describe the maternal behaviours and puppy developments.

For carnivores, the weaning period is quite prolonged, since the offspring has to acquire either hunting ability or social behaviours [37], which is an important skill for a dog in order to adjust to their role as a domestic animal. The events leading to full independence of the puppies from the dam include different interactions. The progression of the mother–puppy relationship described in the first weeks of postpartum from zero to 21 days [1,2,3,28,32] continued during the weaning time. As observed before at the end of the third week [38], all puppies in our study were able to make contact with their mother everywhere inside the maternity area. At the first observation (weeks three/four), as described before [4], although the puppies showed some lack of movement control, they were able to walk backwards and forwards. Over time, suckling became more active, and the puppies were more responsive to initiating the activity. Sucking was a collective activity; when one puppy started to suckle, others followed. Urination and defecation reflexes were present from the first observation time point regardless of stimulation from the mother. All the puppies were autonomous at weeks three/four. Nevertheless, the dam still cleaned the environment after puppies’ elimination. The behaviour of cleaning after each puppy excreta reduced over time but was still observed at the last observation at weeks seven/eight. This could provide one explanation for coprophagy observed in some dogs, since puppies usually learn from the dam’s behaviour. However, although a specific potential link to the mother’s cleaning behaviour was not addressed, a recent study [39] concluded that dogs that were left with their mother for over seven weeks as puppies were not more likely to showing coprophagy than dogs with noncoprophagy behaviours, suggesting this behaviour is not related to a lack of normal mothering.

Another aspect to consider is the variation in the dog breeds between the two groups and the possible impact on some of the observations during the trial. Although anecdotal mothering characteristic differences have been expressed with some breeds being more maternal than others, it is hard to evaluate this feature. The only study looking at the breed effect [2] found a high level of maternal behaviour (calculated through a principal component analysis from seven behavioural variables) favouring Labrador Retrievers as opposed to German Shepherds. Nothing is reported in the literature about maternal styles in the other breeds selected in our study (e.g., English Cockers, American Staffordshire Terriers, Corgis, etc.) that represent the majority (15/25 = 60%) of the bitches in our sample. It seems likely other breed differences probably exist and have just not been studied. In the present study, 63.6% (7/11) of the dams in the placebo group were Labrador Retrievers but none were German Shepherds, whereas, in the ADAPTIL^®^ group, 21.42% (3/14) of the dams were German Shepherds, with no Labradors. Therefore, all else being equal, we would expect the placebo group, based on past research regarding breed differences in maternal styles, to display higher levels of maternal behaviours. However, despite this potential predisposition, we still found greater maternal behaviours in the dogs in the ADAPTIL^®^ group. Conversely, a direct breed effect was observed due to a greater number of puppies in the placebo bitches (mean of 8.0 puppies per litter versus 5.4 in the treatment group). The quantity of puppies’ interactions between puppies and the dam and between puppies and their littermates were de facto influenced by litter sizes, leading to a biased effect towards quantitatively more interactions in the placebo group, despite not being significantly different (Table 6).

The overall temperament of the bitch as perceived by the breeders was a parameter collected in order to check for any imbalances between the groups. This data was important to consider in a study looking at the effects of a soothing product on the maternal behaviours. While most of the selected bitches were qualified as rather calm, which is a good quality for a reproductive bitch, some were perceived as generally more nervous. However, all selected bitches were selected to be reproductive dogs, and none were judged as suffering from chronic stress, since this would prevent them from being used as a breeding stock.

As the puppies became more mobile, the dams started to display rejection signs, such as walking away from puppies as they try to suckle, described as parent–offspring conflict [40,41]. The first signs of rejection were observed at weeks three/four, even if very rare at this stage, and increased by an average of ten times at weeks seven/eight, with no difference observed in relation to the treatment. In association, all lactating-related behaviours were found to show significant differences at each time point. Nursing was significantly longer at weeks three/four and decreased through weeks five/six to the lowest levels in seven/eight-week-old puppies, similar to the findings of several authors [4,14,38,41,42]. Nursing and suckling became, to some extent, antagonist actions, since, on one hand, the puppies wished to suckle, but, alternatively, the dam was not always eager to comply. The reactions of the dams if they were not willing to nurse were variable in our study. During the global period, bitches in the placebo group expressed refusal to nurse mostly by punishment (mean frequency of aggressive growling events per hour recorded over the three time points in the placebo and ADAPTIL^®^ groups were 4.54 and 1.72, respectively). On the other hand, bitches exposed to ADAPTIL^®^ more commonly walked away from the puppies (mean frequency of rejection signs per hour recorded in the placebo and ADAPTIL^®^ groups were 1.52 and 2.04, respectively). The inhibiting bite observed by Wilson [41] was not recorded during the trial, since this behaviour was not very common. However, growling was a common signal of the dam at the puppies, mainly when they tried to suckle. Not all nursing sessions led to conflict reactions. Most of the time, the bitch appeared to be ready for nursing to begin. At the end of the session, puppies could still attempt to suckle or pursue the dam, who, in response, could show rejection or aggressive behaviours. Trying to escape from the puppies’ interactions by moving away was the first signal of separating behaviours. For Rheingold [14], “punishment” was considered a normal maternal behaviour in the bitch, and there was a sequence of situations leading to the punishment. Not all females showed punishing behaviours (some did not at one assessment period, and four bitches–two per group–never expressed it throughout the study), and the time to react to the puppies’ requests were variable as well. The frequency of growling ranged from 0 to 4.5 times per hour at weeks three/four, from 0 to 15.7 at weeks five/six and reached up to 49 times per hour at weeks seven/eight, indicating that the conflictual behaviour was not similar for all the dams [42]. Comparatively, refusing contact was observed up to 3.7, 15.8 and 9.0 times per hour at weeks three/four, weeks five/six and weeks seven/eight, respectively. Growling was the most common and effective way to discourage puppies from all attempts to suckle. On rare occasions, growling was also used to punish a puppy playing too roughly. This agonistic behaviour towards puppies peaked at seven/eight weeks, and no difference was observed between treatments. Although a breed effect could be evoked in the present study, aggressive behaviours such as growling were observed in all breeds represented in the study. Indeed, it was hard to separate the impact of the dam and the offspring in the growling behaviour, since the way the puppies behave towards the bitch or the litter size may prompt this behaviour. Therefore, some mothers were more aggressive towards their offspring because their puppies were more prone to try to suckle and/or more persistent and fierce towards their mother. To better assess the global bitches’ “irritability”, different behaviour signs (rejection, lip-licking and aggressive growling toward pups) were analysed together. The treatment did not lead to significant differences between groups. However, bitches exposed to the pheromone were observed to display less irritability at all time points, suggesting that ADAPTIL^®^ might contribute to the dams being more tolerant.

**Figure 1:**
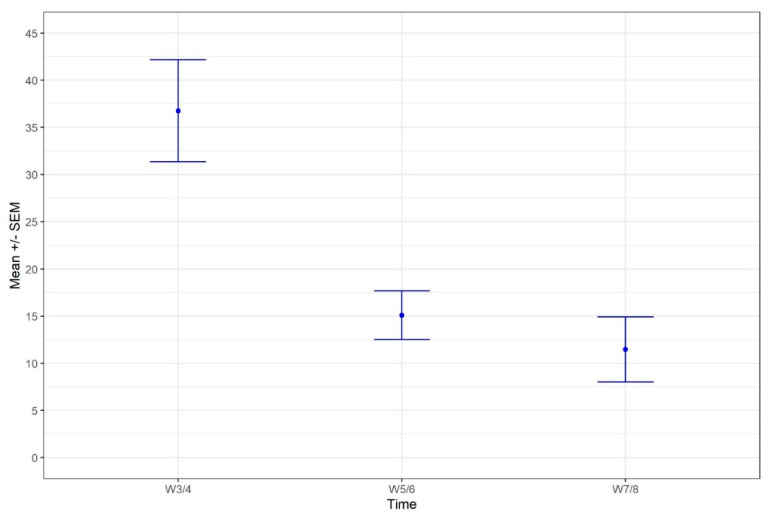
Evolution of the proportion of time dedicated to nurse throughout the weaning period (% of total video observation time, *n* = 25 bitches).

**Figure 2:**
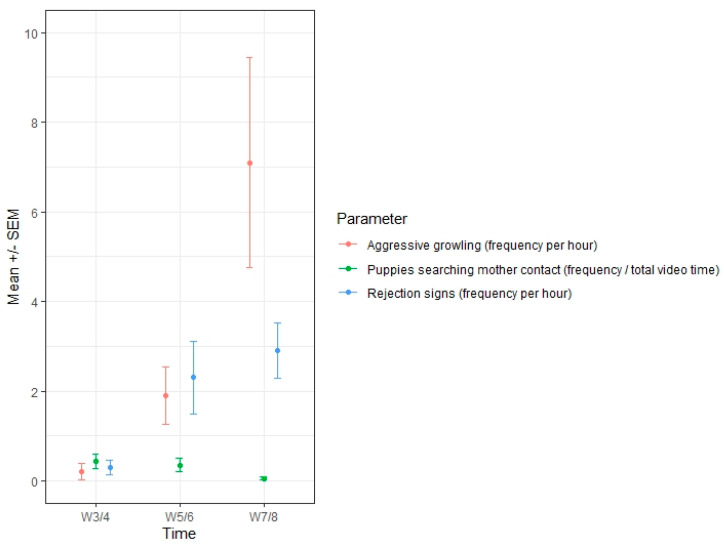
Comparison of the evolutions throughout the weaning period of the frequency of puppies searching contact from the dam as opposed to the dams’ reactions by aggressive growling or rejection (*n* = 25 bitches).

**Figure 3:**
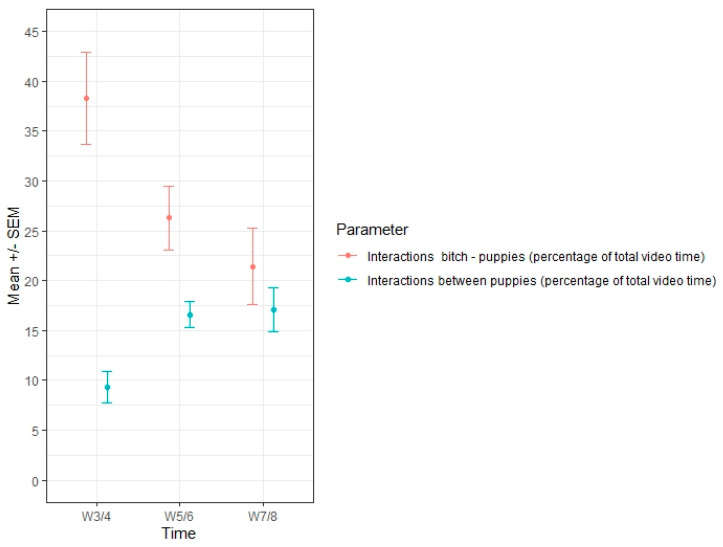
Proportional time dedicated to interactions from W3/4 to W7/8 postpartum (*n* = 25 bitches). Comparison of the opposite evolutions throughout the weaning period of interactions amongst puppies and interactions between the dam and the puppies.

As suggested in other mammals [43], the puppies actively participate in the weaning process by reducing the duration of suckling (being more skilled at the task with age). Thereby, it seems to be a synchronous experience: dams have more difficulties staying still for nursing at the same time as the puppies are able to suckle faster, leading to a steady decrease in the duration of nursing (Figure 1 and Figure 2). A similar switch of behaviours was also observed for caregiving from the bitch as opposed to refusing contact with her puppies and a reduction in contact-seeking by the puppies. Nevertheless, the puppies continued to try to interact with the dam, with increased aggression observed at weeks seven/eight (Figure 2). All the behaviours seemed to follow a logical path: as the puppies got older, they spent less time pestering their mother and more time interacting with their littermates (Figure 3). However, even at the last time point (weeks seven/eight), the puppies apparently still preferred to interact with their mother than their siblings (as expressed by the respective percentages of time spent to each interaction; Figure 3), and they still tried to suckle even though, at a nutritional level, they were no longer reliant on the dam’s milk. The persistence in this behaviour might reflect a social effect of suckling on the puppy’s development [13].

The position of nursing also changed as the puppies got older (all bitches together). During weeks three/four, 7.8% of nursing was carried out in a standing and 79% in a lying down position, as opposed to 16.4% in a lying position and 70.1% standing at weeks seven/eight. Moreover, a difference was observed between treatments, with ADAPTIL^®^ bitches nursing significantly more in a lying position. However, the number of puppies in the litter could influence this posture. In the case of a large litter, the only way to allow all the puppies to nurse at the same time is by standing, as all the nipples then become available. Since the litter sizes of the ADAPTIL^®^ bitches were significantly smaller, this criterion (number of puppies per bitch) was integrated as a covariate in the statistical models and was shown to be not significant on the nursing position (*p* = 0.1). Another hypothesis to explain the high frequency of nursing in a lying down position could be that bitches exposed to the pheromone were more relaxed. To quantify the levels of stress, puppies’ behaviours were observed during the three time points. No single specific behaviour could be considered as an indicator of stress; therefore, they were combined for each time point. As suggested before, maternal separation can lead to distress behaviours from the third week postpartum [44,45,46]. In addition, early maternal separation at six weeks old has a negative effect on the physical condition of the puppy and impairs the bonding process of puppies with humans when compared with a later weaning time at 12 weeks [47]. The diversity of behaviours during the weaning period was consistent with other authors [4,14,41]. Based on the video recording, the activities were somewhat cyclical; once the puppies woke up, they would search the dam to suckle, play and then sleep again. Sleeping was the most common activity of the puppies at all time points, and no difference was observed between treatments. The most common puppy-to-puppy interaction was mutual mouthing. The first playing behaviour was observed at weeks three/four and increased over time. Bonded relationships, defined here as close interactions, were observed amongst the puppies and, also, between the dam and her offspring in all cases, independently of the number of littermates. Since puppies were not observed individually, we cannot affirm if there was any preference amongst the littermates or if they formed dyads or small-established groups. However, in our observations, once the mother was present, the strongest interest of the puppies was towards her, as previously reported [14].

Regarding breeders’ perceptions on maternal care, all VAS criteria assessing, separately, bitches and their litters showed significant differences favouring the ADAPTIL^®^ group. Breeders’ perceptions were unbiased, since they were blinded towards the treatment their dogs were exposed to during the weaning period and could not detect it (the canine pheromone analogue has no intrinsic odour and is species-specific; humans, for example, cannot perceive it [18]). The analysis of the full study period, including weeks three/four, since the diffusers were placed before this first assessment time, allowed conclusions on the global benefits on the weaning period. In addition, this age is the age limit for the stress-hyporesponsive period (SHRP), suggesting that no difference should be observed between the two groups at weeks three/four [38]. It should be noted that all VAS criteria scored quite high (between 6.9 and 9.0) at all time points, meaning positive qualitative assessments of the bitches and puppies’ reactions and interactions. Considering that breeders scored in blinded conditions, this makes the significant differences observed between groups during the full study period even more powerful within this narrow range. Coping with the weaning period is an important goal to breeders. The results suggested that breeders perceived a strong effect of the product, favouring a positive attitude from mothers towards the puppies and a positive behavioural development of the puppies. Since the reduction in both the amount of milk and time spent to nurse, as well as the levels of interactions between mother and offspring during weaning, can affect the behaviours of the puppies [41], a smoother transition during this period might bear a positive effect. In relation to the difference observed in the “resting time” slightly higher for the bitches exposed to ADAPTIL^®^, no physiological explanation can be drawn from this tendency, since the bitches were not under video surveillance all the time. Therefore, the presence of ADAPTIL^®^ during the first weeks of life seems to contribute to improving the ability of puppies to cope better with frustrations. Combined with the previous study assessing ADAPTIL^®^ during peripartum [28], those results suggest potential benefits for breeders in providing a dog-appeasing pheromone to their dogs from whelping to weaning. However, how the early life experiences will influence the dogs’ lives later on is still unclear. Although it has been suggested that excessive mothering can lead to poorer outcomes for the puppies in the guide dog population, probably because they are less exposed to challenging/stressful events [48], the same might not be applicable for the general dog population. Not only are the expectations for a guide dog different, the selection process is unique as well. Therefore, at this point, it is not possible to say if reduced stress around the weaning time could have a negative impact in the long term. Most likely, a more serene environment throughout the weaning period is beneficial. A possible way to explore this hypothesis would be to design a future follow-up study assessing how the puppies from the litters involved in the current study develop behaviourally.

## 5. Conclusions

In recent years, there has been increased attention to better understand the major aspects controlling the behaviours and temperaments in dogs. The reason behind this interest is not only to have clues that could improve the daily lives of pet dogs and their respective owners but to develop better methods for selecting working dogs for human service as well. The weaning time is part of the critical period of socialisation. Therefore, perhaps an easier and less frustrating time might improve the relationship between dam and littermates, which could potentially lead to better cognitive behaviours of puppies in their later development. In this study, ADAPTIL^®^ had an impact on the nursing position, the avoidance behaviour of the dam during the weaning process (bitches were more tolerant) and the puppies’ coping abilities during this transitional and frustrating period. The breeders on the mothers’ attitudes towards their litters, as well as the puppies’ reactions, also perceived the benefits of the treatment.

## Figures and Tables

**Table 1:** Classification of maternal and puppy behaviours assessed through video recording throughout the weaning period.

Items	Behaviour	Definition
Maternal behaviour State events	Inside the maternity area	Bitch present in the maternity area where camera was set
	Contact with puppies	Close proximity (body contact) of the bitch (in sitting or lying down position) to at least one puppy
	Nursing and posture	At least one puppy attached to the nipple and the position of the bitch during the nursing (sitting, standing or lying down) was recorded
	Oro-nasal interaction	Licking, grooming and/or sniffing a puppy
Maternal behaviour Point events	Rejection sign	Bitch walking away from the puppies
	Aggressive Growling	Any signs of aggression towards at least one puppy
	Lip-licking	Bitch passing her tongue over the upper lip
Puppy behaviour State events	Interaction with the dam	At least one puppy interacting with the dam (jumping to or climbing on mother, walking around her, seeking contact, etc.)
	Interaction with another puppy	At least two puppies interacting with each other (playing, mouthing, etc.)
	Searching-looking signs	At least one puppy actively seeking the dam when she leaves the area (staying close to the exit, whining, etc.)
	Wandering around	Puppy roaming from one place to another
	Sleeping	At least one puppy sleeping
Puppy behaviour Point events	Yawning	Involuntarily opening of the mouth of a puppy
	Barking	At least one puppy making sound
	Scratching	At least one puppy scratching its body with their paws
	Aggressive growling	A puppy growling at another puppy (although it could be part of a normal interaction, this behaviour was considered as a sign of belligerence).

**Table 2:** Descriptive demographics on the selected bitches and kennels.

Characteristics	ADAPTIL^®^	Placebo
Bitches (number)	14	11
Breed	English Cocker (5) German Shepherd (3) Spitz (2) Australian Cattle dog (1) Welsh Corgi (1) Dachshund (1) Golden Retriever (1)	Labrador Retriever (7) American Staffordshire Terrier (2) Golden retriever (2)
Mean age in years (min–max)	3.1 (1.0–5.0)	3.8 (2.0–8.0)
Litter size: mean number of puppies per bitch (min–max)	5.4 (3–9)	8.0 (5–11)
Parity	Primiparous = 4/14 (28.6%) Multiparous = 10/14 (71.4%)	Primiparous = 4/11 (36.4%) Multiparous = 7/11 (63.6%)
General temperament	Calm = 8/12 (66.7%) Nervous = 4/12 (33.3%)	Calm = 8/11 (72.7%) Nervous = 3/11 (27.3%)
Breeding kennels (number)	4	4
Size of the kennel	Small= 1/4 (25.0%) Medium = 2/4 (50.0%) Large = 1/4 (25.0%)	Small= 1/4 (25.0%) Medium = 3/4 (75.0%)
Number of different breeds raised in the kennels	2 breeds = 2/4 (50.0%) 3 breeds = 1/4 (25.0%) 10 breeds = 1/4 (25.0%)	2 breeds = 3/4 (75,0%) 3 breeds = 1/4 (25.0%)
Housing for weaning period	Single box = 11/14 (78.6%) Collective = 3/14 (21.4%)	Single box = 3/11 (27.3%) Collective = 8/11 (72.7%) →5/8 (62.5%) transferred to single boxes at week (W)5/6

**Table 3:** Evolution of the proportion of time spent by bitches to nurse throughout the weaning period (percentage of total video-recorded time).

Items	Total (*N* = 25)	ADAPTIL^®^ (*N* = 14)	Placebo (*N* = 11)
W3/4	36.8%	31.5%	43%
W5/6	15.1%	13.5%	17.2%
W7/8	11.5%	7.4%	15.9%

**Table 4:** Evolution of the nursing position of the bitches throughout the weaning period (percentage of total time spent to nurse + SD).

Items	Lying Down	Standing	Sitting
W3/4	79.0 (±30.0)	7.8 (±12.2)	13.4 (±25.3)
W5/6	45.8 (±37.6)	31.2 (±33.1)	11.0 (±22.3)
W7/8	16.4 (±30.1)	70.1 (±36.1)	13.5 (±23.4)

**Table 5:** Nursing position of the bitches during the full weaning period (percentage of total time spent to nurse + SD).

Items	Total (*N* = 25)	ADAPTIL^®^ (*N* = 14)	Placebo (*N* = 11)	*p*-Value
Nursing lying down	66.3 (±27.4)	75.8 (±25.0)	54.1 (±26.4)	0.007
Nursing standing	21.2 (±18.5)	15.0 (±12.4)	29.2 (±22.2)	0.011
Nursing sitting	12.5 (±20.3)	9.2 (±21.5)	16.6 (±18.6)	0.037

**Table 6:** Behavioural signs observed in puppies and bitches through video recording throughout the weaning period at each time point and during the full weaning period.

Behavioural Signs Observed in Puppies (Mean +/− SD)	Time Period	Total (*N* = 25)	Placebo (*N* = 11)	ADAPTIL^®^ (*N* = 14)	*p*-Value
Sleeping (percentage of total video time)	All periods combined	82.4 (±6.3)	83.4 (±5.6)	81.5 (±7.0)	0.461
	W3/4	89.7 (±7.6)	91.0 (±5.3)	88.6 (±9.1)	0.446
	W5/6	82.8 (±8.5)	80.6 (±8.6)	84.5 (±8.2)	0.264
	W7/8	71.8 (±16.2)	74.3 (±16.5)	69.9 (±16.4)	0.532
Latency time when bitch comes back into maternity area (time in seconds)	All periods combined	15.5 (±22.3)	13.6 (±28.4)	17.0 (±17.7)	0.196
	W3/4	14.5 (±20.9)	11.8 (±23.2)	16.9 (±20.2)	0.283
	W5/6	4.5 (±6.8)	2.2 (±2.9)	6.6 (±8.6)	0.259
	W7/8	2.8 (±7.1)	4.2 (±9.7)	1.1 (±0.9)	0.326
Searching mother (frequency/total video time)	All periods combined	0.29 (±0.41)	0.28 (±0.38)	0.30 (±0.46)	0.976
	W3/4	0.43 (±0.79)	0.52 (±0.93)	0.35 (±0.68)	0.560
	W5/6	0.35 (±0.73)	0.18 (±0.43)	0.48 (±0.90)	0.488
	W7/8	0.05 (±0.17)	0.04 (±0.14)	0.06 (±0.20)	1.000
Yawning + Lip-licking + Barking + Scratching + Aggressive growling + Searching mother (frequency/total video time)	All periods combined	11.07 (±4.96)	14.36 (±4.85)	8.49 (±3.31)	0.003
	W3/4	11.1 (±7.6)	14.9 (±8.9)	7.9 (±4.6)	0.035
	W5/6	9.7 (±6.1)	11.7 (±6.2)	8.1 (±5.7)	0.145
	W7/8	11.9 (±8.0)	14.0 (±10.0)	10.2 (±5.5)	0.241
Interactions between puppies (percentage of total video time)	All periods combined	14.0 (±5.8)	14.7 (±7.2)	13.4 (±4.7)	0.589
	W3/4	9,3 (7,9)	11,3 (8,2)	7,7 (7,6)	0.247
	W5/6	16,6 (6,5)	18,4 (5,3)	15,3 (7,2)	0.238
	W7/8	17,1 (11,0)	14,9 (13,4)	18,7 (9,0)	0.425
**Behavioural signs observed in bitches**		**Total (*N* = 25)**	**Placebo (*N* = 11)**	**ADAPTIL^®^ (*N* = 14)**	***p*-value**
Licking puppy (frequency per hour of total video time)	All periods combined	3.1 (±1.9)	2.5 (±1.4)	3.6 (±2.1)	0.262
	W3/4	4.3 (±2.1)	3.3 (±2.2)	5.2 (±1.7)	0.025
	W5/6	2.8 (±2.6)	2.7 (±2.6)	3.0 (±2.7)	0.904
	W7/8	1.9 (±2.6)	1.5 (±2.0)	2.2 (±3.2)	0.750
Interactions between bitch and puppies (percentage of total video time)	All periods combined	28.4 (±15.1)	30.7 (±19.6)	26.6 (±10.7)	0.545
	W3/4	38.3 (±23.1)	41.2 (±29.9)	35.9 (±16.4)	0.593
	W5/6	26.3 (±16.1)	27.0 (±15.3)	25.7 (±17.2)	0.848
	W7/8	21.4 (±19.2)	24.6 (±27.0)	18.8 (±9.3)	0.668
Aggressive growling towards puppies (frequency per hour of total video time)	All periods combined	1.8 (±2.4)	2.5 (±3.3)	1.3 (±1.3)	0.681
	W3/4	0.8 (±3.1)	1.4 (±4.5)	0.3 (±0.6)	0.475
	W5/6	4.4 (±6.0)	5.3 (±8.1)	3.6 (±3.7)	0.822
	W7/8	10.0 (±18.0)	15.2 (±24.4)	5.3 (±7.4)	0.395
Rejection signs from bitches + lip-licking + aggressive growling towards pups (frequency/total video time)	All periods combined	5.7 (±3.1)	5.8 (±3.7)	5.5 (±2.8)	0.845
	W3/4	3.2 (±2.5)	3.9 (±3.2)	2.6 (±1.7)	0.239
	W5/6	5.3 (±4.0)	4.2 (±3.7)	6.1 (±4.2)	0.267
	W7/8	9.9 (±11.9)	12.2 (±16.7)	7.7 (±4.4)	0.735

Note: Behavioural signs in bitches are expressed based on the time spent by bitches inside the maternity area (where cameras were located), while puppy behaviours are expressed based on the full video-recording time, since some behaviours may occur in the presence or in the absence of the bitches.

**Table 7:** Distribution of the visual analogue scale (VAS) scores assessing bitches and puppies’ interactions according to time point (*N* = 25). Mean values out of a total of 10 + standard deviation.

VAS Criteria	W3/4	W5/6	W7/8
**Mother parameters**			
Bitch calm when puppies try to suckle	8.8 (±2.3)	6.9 (±3.3)	7.6 (±2.9)
Bitch patient, never aggressive towards puppies	9.2 (±1.9)	8.1 (±2.7)	8.5 (±1.8)
Global relationship to puppies, mother attitude	8.9 (±1.9)	7.5 (±3.6)	8.9 (±1.6)
**Puppies parameters**			
Puppies not crying nor looking for mother when away	8.0 (±2.2)	8.2 (±2.4)	8.5 (±1.8)
Peaceful interaction between puppies without competition	9.0 (±1.1)	8.4 (±2.3)	8.1 (±2.2)

**Table 8:** Distribution of the visual analogue scale (VAS) scores according to treatment, all times combined (*N* = 25). Mean values out of a total of 10 + standard deviation.

VAS Criteria	Placebo (*N* = 11)	ADAPTIL^®^ (*N* = 14)	*p*-Value (Wilcoxon Test)
**Mother parameters**			
Bitch calm when puppies try to suckle	6.2 (±1.8)	9.1 (±0.9)	0.001
Bitch patient, never aggressive towards puppies	7.7 (±1.7)	9.3 (±0.9)	0.025
Global relationship to puppies, mother attitude	7.2 (±2.0)	9.4 (±0.6)	0.016
**Puppies parameters**			
Puppies not crying nor looking for mother when away	6.9 (±1.3)	9.3 (±0.4)	<0.001
Peaceful interaction between puppies without competition	7.8 (±1.7)	9.0 (±0.8)	0.055
